# „In cabin rapid sequence induction“: Erfahrung aus der alpinen Luftrettung zur Verkürzung der Prähospitalzeit

**DOI:** 10.1007/s00101-021-00933-8

**Published:** 2021-03-08

**Authors:** Jürgen Knapp, Philipp Venetz, Urs Pietsch

**Affiliations:** 1 Klinik für Anästhesiologie und Schmerztherapie, Universitätsspital Bern, Freiburgstraße, 3010 Bern, Schweiz; 2 Air Zermatt AG, Zermatt, Schweiz; 3 Zentrum für Intensivmedizin, Luzerner Kantonsspital, Luzern, Schweiz; 4 Klinik für Anästhesiologie, Intensiv‑, Rettungs- und Schmerzmedizin, Kantonsspital St. Gallen, St. Gallen, Schweiz; 5 Universitäres Notfallzentrum, Inselspital, Universitätsspital Bern, Bern, Schweiz

**Keywords:** Rettungshubschrauber, Anästhesie, Airway-Management, „Standard operating procedure“, Patientensicherheit, Emergency helicopters, Anesthesia, Airway management, Standard operating procedure, Patient safety

## Abstract

Das Überleben von Schwerverletzten ist von der schnellen und effizienten prähospitalen Versorgung abhängig. Die Zeit vom Unfallereignis bis zum Eintreffen des Patienten im Schockraum konnte leider trotz aller Bemühungen der vergangenen Jahrzehnte und trotz des immer dichteren Netzes an Rettungshubschraubern (RTH), bislang nicht relevant verkürzt werden. Ein gewisser Anteil der Schwerverletzten benötigt bereits prähospital eine Narkoseeinleitung (typischerweise als „rapid sequence induction“, RSI). Durch die medizinischen und technischen Fortschritte der Videolaryngoskopie sowie der im deutschsprachigen Raum eingesetzten Luftrettungsmittel erscheint die Möglichkeit, unter bestimmten Bedingungen die Narkoseeinleitung und das Airway-Management in der Kabine des RTH – also während des Transports – durchzuführen, als mögliche Option, um die Prähospitalzeit zu verkürzen. Für die sichere Durchführung sind die im vorliegenden Beitrag behandelten Aspekte elementar. Beispielhaft wird ein Prozedere vorgestellt, das sich seit geraumer Zeit bewährt hat. Die „in cabin RSI“ sollte allerdings nur von zuvor trainierten Teams bei Vorliegen einer klaren „standard operating procedure“ durchgeführt werden.

## Abstract (en)

The survival of the severely injured is dependent on the rapid and efficient prehospital treatment. Despite all efforts over the last decades and despite an improved network of rescue helicopters, the time delay between the accident event and admission to the trauma room could not be reduced. A certain proportion of the severely injured need induction of anesthesia even before arrival in hospital (typically as rapid sequence induction, RSI). Due to the medical and technical progress in video laryngoscopy as well as in the means of air rescue used in German-speaking countries, under certain conditions the possibility to carry out induction of anesthesia and airway management in the cabin of the rescue helicopter, i.e. during the transportation, seems to be a possible option to reduce the prehospital time. The aspects dealt with in this article are elementary for a safe execution. A procedure that has been tried and trusted for some time is presented as an example; however, the in-cabin RSI should only be carried out by pretrained teams using a clear standard operating procedure.

## Fallvignette

Die Alarmierung Ihres Teams als Besatzung eines Rettungshubschraubers (RTH) erfolgt zu einem Verkehrsunfall mit Beteiligung eines Fahrradfahrers. Der Unfallort befindet sich auf einer Passstraße 25 bis 30 Flugminuten entfernt von der nächstgelegenen Klinik der Maximalversorgungsstufe. Bei Eintreffen am Einsatzort finden Sie eine ca. 30-jährige Rennradfahrerin vor, die offensichtlich bei der Abfahrt in einer Haarnadelkurve ohne Fremdeinwirkung gestürzt und laut Augenzeugen mit hoher Geschwindigkeit gegen den Pfosten der Leitplanke geprallt ist. Der Helm wurde von Augenzeugen bereits abgenommen. Ihre initiale Untersuchung ergibt folgende Befunde:

- „Airway“ (A): frei, Patientin aber zeitweise schnarchend, daher gefährdet; Halswirbelsäule durch ausgebildete Ersthelfer manuell immobilisiert,
- „Breathing“ (B): unauffällig,
- „Circulation“ (C): Radialispuls kräftig und regelmäßig tastbar, normale Rekapillarisierungszeit, keine offensichtlichen Blutungen,
- „Disability“ (D): Score der Glasgow Coma Scale 6 (Augenöffnen 1, verbale Antwort 1, motorische Antwort 4), Pupillen isokor und seitengleich lichtreagibel, bewegt auf Schmerzreiz beide Arme, Beine ohne motorische Reaktion,
- „Exposure, environment“ (E): keine äußeren Blutungen, Thorax stabil, Abdomen weich, Beckentrauma von der Kinematik her möglich, keine Frakturzeichen an den Extremitäten, ausgedehnte Schürfwunden an beiden Unterarmen und den Knien.

Sie ziehen die Patientin achsengerecht unter der Leitplanke hervor, lagern sie zur Zeitersparnis in diesem Arbeitsschritt bereits schonend „en bloc“ auf einer Vakuummatratze, immobilisieren mit zusätzlichen „head blocks“ auch die HWS und schließen die Beckenschlinge. Beim nochmaligen Blick in die Pupillen fällt Ihnen nun eine leichte Pupillendifferenz (rechts > links) auf. Des Weiteren ist die Herzfrequenz von initial 88/min auf 123/min angestiegen, der Blutdruck, aktuell gemessen, beträgt 100/66 mm Hg. Da Sie mit dem RTH das einzige Einsatzmittel vor Ort sind, konnten bis auf Monitorisierung, Bergung, Lagerung und Immobilisierung bisher keine weiteren Maßnahmen ergriffen werden. Sie entscheiden sich daher zum sofortigen Verladen der Patientin in den RTH sowie zu Narkoseeinleitung und Atemwegssicherung während des Flugs und verlassen die Einsatzstelle bereits nach 7 min. Während Sie sich am Kopfende der Patientin positionieren, die „Airway-Tasche“ und das Videolaryngoskop vorbereiten sowie den Trachealtubus (Innendurchmesser 7,0 mm) bereitlegen, hat der Notfallsanitäter einen i.v.-Zugang am rechten Handrücken der Patientin etabliert. Sie sprechen sich kurz ab, dass Sie nun noch Midazolam und Fentanyl aufziehen, der Notfallsanitäter das Rocuronium vorbereitet und besprechen bereits die für die Narkoseeinleitung zu applizierenden Dosen der Medikamente. Noradrenalin (10 µg/ml) wird standardmäßig fertig aufgezogen mitgeführt. Sämtliche Spritzen werden auf einem kleinen Tableau abgelegt. Narkoseeinleitung und tracheale Intubation gelingen problemlos. Sie übergeben die Patientin 33 min nach dem Start an der Unfallstelle intubiert und seitengleich beatmet mit klinischen Zeichen des hämorrhagischen Schocks (Herzfrequenz 140/min, Blutdruck 100/70 mm Hg unter repetitiver bolusweiser Gabe von 10 µg Noradrenalin) und einer nun deutlichen Pupillendifferenz im Schockraum des überregionalen Traumazentrums. Die Diagnostik dort ergibt eine instabile Beckenringfraktur und ein schweres Schädel-Hirn-Trauma mit großem linksseitigem akutem Subduralhämatom und Mittellinienverlagerung. Die Patientin wird umgehend zur Hemikraniektomie und parallelen Anlage einer Beckenzwinge in den OP verbracht. Bereits auf dem Weg dorthin wird bei zunehmender hämodynamischer Instabilität der Patientin mit der notfallmäßigen Transfusion von Erythrozytenkonzentraten (da noch keine Kreuzprobe vorliegt: Blutgruppe 0, Rhesusfaktor negativ) begonnen. Insgesamt müssen 6 g Fibrinogen, 12 Erythrozytenkonzentrate, 6 Beutel gefrorenes Frischplasma (FFP) und ein Thrombozytenhochkonzentrat appliziert werden.

## Hintergrund

**Figure 1:**
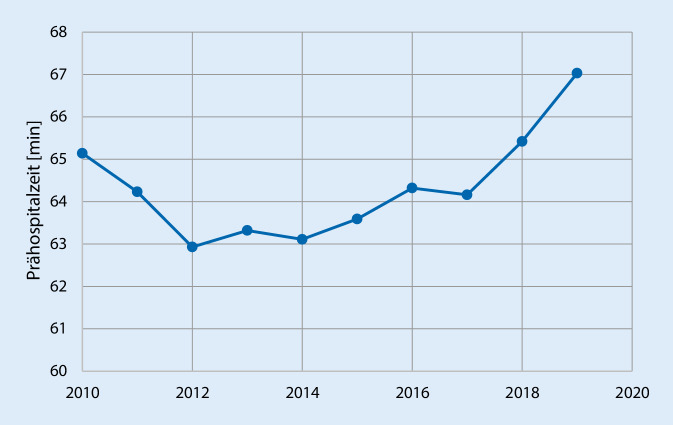

Das Überleben von Schwerverletzten ist von einer schnellen und effizienten prähospitalen Versorgung abhängig. Für eine verlängerte Prähospitalzeit von Traumapatienten ist eine Assoziation zur erhöhten Sterblichkeit insbesondere bei Vorliegen eines Schädel-Hirn-Traumas nachgewiesen [1–3]. Trotz aller Bemühungen der vergangenen Jahrzehnte und trotz des immer dichteren Netzes an Rettungshubschraubern (RTH) konnte die Prähospitalzeit (definiert als die Zeit vom Unfallereignis bis zur Aufnahme im Schockraum) nicht relevant verkürzt werden. So beträgt sie gemäß der Auswertung des Traumaregisters der Deutschen Gesellschaft für Unfallchirurgie (DGU) für schwer verletzte Patienten seit 2010 zwischen 63 und 67 min und scheint in den vergangenen Jahren tendenziell eher zuzunehmen (Abb. 1; [4]).

Ein gewisser Anteil der Schwerverletzten benötigt bereits prähospital eine Narkoseeinleitung (typischerweise als „rapid sequence induction“, RSI). Die prähospitale RSI ist mit einem deutlich höheren Komplikationsrisiko verbunden als innerklinisch und sollte entsprechend aktueller Leitlinien unter möglichst optimalen äußeren Bedingungen, möglichst sicher und dennoch mit dem geringsten möglichen Zeitverlust durchgeführt werden [5, 6]. Üblicherweise werden solche Patienten (meist abhängig von den äußeren Bedingungen) zunächst im Rettungswagen (RTW) oder direkt an der Unfallstelle versorgt und intubiert, bevor sie in den RTH verbracht und transportiert werden. Gemäß einer Auswertung aus dem Traumaregister der DGU kann allein für die prähospitale Narkoseeinleitung und Intubation ein Zeitaufwand von ca. 11 min geschätzt werden [7]. Die Auswertung von eigenen Daten ergibt einen Zeitbedarf von im Median 13 min (Interquartilenabstand: 9–15 min; unveröffentlichte eigene Daten). In der alpinen Luftrettung kommen zu den bekannten Gefahren und Herausforderungen der prähospitalen RSI oft noch widrige Umgebungsbedingungen wie extreme Kälte, Wind, Niederschläge, helles Licht auf einem Gletscher, Dunkelheit oder exponiertes Gelände erschwerend hinzu. Die Möglichkeit, die RSI in einem RTW durchzuführen, besteht hier meist nicht. In solchen Fällen musste der Patient bisher oft nach einer Windenrettung an einem Zwischenlandeplatz außerhalb des Helikopters versorgt und ggf. eine RSI durchgeführt werden, bevor er dann wieder für den Transport in den RTH verladen wurde [8, 9].

## Fortschritte in der prähospitalen Notfallmedizin

**Figure 2:**
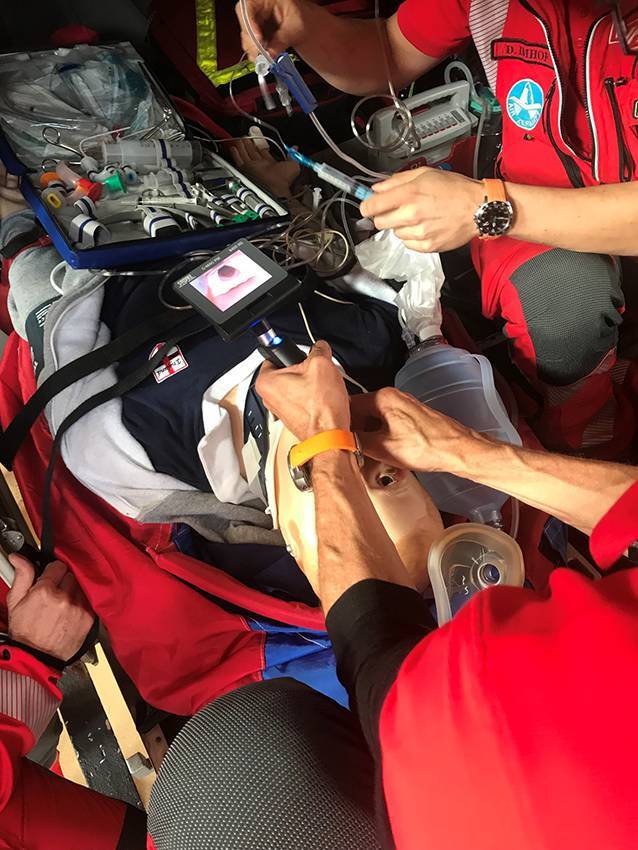

Durch die technischen Fortschritte der vergangenen Jahre ergeben sich neue Möglichkeiten, die Prähospitalzeit bei intubationspflichtigen Patienten deutlich zu verkürzen. Zum einen ist inzwischen die Videolaryngoskopie auch in der prähospitalen Notfallmedizin weitgehend etabliert und sollte gemäß den S1-Leitlininen zur prähospitalen Narkoseeinleitung auch standardmäßig eingesetzt werden [6]. Die Verwendung dieser Technik erhöht nicht nur den Intubationserfolg und die Rate der im ersten Versuch erfolgreichen Intubationsmanöver („first pass success“), sondern erfordert auch nicht mehr zwingend, dass die Blickachse des Intubierenden exakt in eine Linie zur Stimmbandebene des Patienten gebracht wird. Zum anderen sind die Kabinen der im deutschsprachigen Raum eingesetzten Luftrettungsmittel in den letzten Jahren deutlich geräumiger geworden. Die Kabinen moderner in der Luftrettung eingesetzter Hubschrauber sind inzwischen sehr geräumig und bieten meist eine nutzbare Kabinenlänge von deutlich über 2,5 m (Tab. 1). Dies erlaubt bei üblicher Innenausstattung einem Teammitglied, bequem eine knieende oder sitzende Position hinter dem Kopf des in der Kabine liegenden Patienten einzunehmen (Abb. 2).

**Table 1:** 

Hubschraubertyp	Deckenhöhe im Bereich des Patientenkopfes^a^ (cm)	Kabinenlänge im Bereich des Stretchers^a^ (cm)	Kabinenvolumen (laut Hersteller) (m^3^)
BK 117	129	250	4,87
EC 135 bzw. H 135	125	260	4,80
AW-109 SP	90	215	4,05
H 145	127	344	6,00
Bell 429	120	310	5,78
AW-169 FIPS	127	277	6,28

^a^Je nach Innenausbau der Kabine können die Maße von den angegeben Daten abweichen. Die hier angegeben Daten entsprechen den Ausbauvarianten, die den Autoren zur Messung zur Verfügung standen

Somit erscheint die Möglichkeit, unter bestimmten Bedingungen die Narkoseeinleitung und das Airway-Management in der Kabine des RTH – also bereits während des Transports – durchzuführen, als mögliche Option, um die Prähospitalzeit zu verkürzen. In Gebieten, in denen RTH häufig als alleiniges Rettungsmittel eingesetzt werden (alpine Regionen, sehr ländliche Regionen etc.), bietet zudem oft nur die Kabine des Hubschraubers einen entsprechenden Schutz vor Witterungseinflüssen und einer Auskühlung des Patienten.

## Studien zur „in cabin rapid sequence induction“

Bisher existieren nur wenige Studien bezüglich der „in cabin RSI“. In der Literatur wird abhängig vom Zeitpunkt zwischen Intubationen am Unfallort („on scene“), in der Hubschrauberkabine vor dem Start der Triebwerke („preflight“) und Intubationen während des Flugs („en route“) unterschieden. Die bisher vorliegenden Daten sind allerdings sehr heterogen, da die Zusammensetzung der Teams nicht vergleichbar ist und unterschiedliche Helikoptertypen mit entsprechend nichtvergleichbaren Platzverhältnissen eingesetzt werden. Einen Überblick über die aktuell vorliegenden Studien unter realen Einsatzbedingungen bietet Tab. 2.

**Table 2:** 

Studie (Erstautor, Jahr)	Team	Hubschraubertyp	Erfolgsraten	
			„En route“	„On scene“
Mishark 1992 [10]	Flight nurses	BK 117	79 % (57/72)	94 % (63/64)
Harrison 1997 [11]	1 flight nurse, 1 paramedic	BK 117	94 % (113/120)	98 % (118/120)
Slater 1998 [12]	2 flight nurses	BK 117	98 % (100/102)	96 % (179/186)
Thomas 1999 [13]	1 flight nurse, 1 paramedic	BK 117, AS 365N2 Dauphin	96 % (235/246)	96 % (112/117)
McIntosh 2008 [14]	1 flight nurse, 1 paramedic	BK 117, Bell 206 (2,1 m^3^), Bell 407 (2,4 m^3^), Bell 430 (4,5 m^3^)	89 % (57/64)	95 % (595/627)
Maeyama 2020 [15]	2 HEMS physicians, 1 nurse	EC 135	98 % (189/192)	97 % (179/184)

^a^Simulator- oder „Manikin“-Studien wurden nicht berücksichtigt

*HEMS* „helicopter emergency medical system“

Diese Ergebnisse sind aus verschiedenen Gründen nicht auf die aktuellen Verhältnisse in der Luftrettung Mitteleuropas zu übertragen. Bis auf die Arbeit von Maeyama et al. wurden alle Studien mit konventioneller direkter Laryngoskopie durchgeführt. Auch die eingesetzten Hubschraubertypen (überwiegend BK 117) bieten weniger Raum und Kabinenlänge als die meisten inzwischen im deutschsprachigen Raum üblichen RTH. Eine aktuelle kleine „Manikin“-Studie aus Skandinavien, die in einem RTH vom Typ H 145 unter Einsatz von Videolaryngoskopie durchgeführt wurde, kam zu dem Schluss, dass eine „in cabin RSI“ mit einem Team, bestehend aus Arzt und „paramedic“, unter „Preflight“-Bedingungen (also bei abgestelltem Triebwerk) genauso schnell und erfolgreich durchgeführt werden kann wie eine Intubation außerhalb des Hubschraubers (100 %ige Erfolgsrate, *n* = 14, [16]).

## Prozedere

Die „in cabin RSI“ während des RTH-Transports ist gegenüber dem konventionellen Vorgehen – also der Narkoseeinleitung und Intubation am Unfallort – aber auch mit gewissen Nachteilen und Risiken verbunden, die im Rahmen einer Nutzen-Risiko-Abwägung berücksichtigt werden müssen:

- Ein Zugang zum (erwachsenen) Patienten ist in der RTH-Kabine nicht von allen Seiten möglich, sondern nur vom Kopfende und – je nach Ausbau der Kabine – entweder von der linken oder rechten Patientenseite.
- Bei einem Verschmutzen der Kameralinse (z. B. durch Blut oder Schleim) ist ein Wechsel zur direkten Laryngoskopie möglicherweise erschwert, und das Laryngoskop muss zur Reinigung der Linse entfernt werden.
- Die Auskultation des Patienten ist während des Flugs unmöglich.
- Die Kommunikation innerhalb des Teams ist nur über das Kabinenkommunikationssystem des RTH („Interkom“) möglich.

Bei der Air Zermatt besteht die Crew, vergleichbar vieler anderer europäischer Luftrettungsorganisationen, aus einem Facharzt für Anästhesie mit jahrelanger Expertise im Airway-Management und einem erfahrenen Rettungssanitäter. Aufgrund der oft widrigen Umgebungsbedingungen im hochalpinen Einsatzgebiet werden mit sehr guter Erfahrung nach der Ausbildung unter Simulationsbedingungen bereits seit geraumer Zeit invasive Maßnahmen sowie auch die Narkoseeinleitung und tracheale Intubation innerhalb der Hubschrauberkabine durchgeführt (sowohl „preflight“ als auch „en route“; Abb. 2).

Folgendes Prozedere ist hierfür etabliert: Die Modultasche zum Airway-Management („Airway-Tasche“) mit allem notwendigen Material für die tracheale Intubation wird entweder auf dem Patienten oder rechts neben dem intubierenden Teammitglied auf dem Kabinenboden abgelegt. Der Intubierende kniet oder hockt, wie bei der Intubation eines auf dem Boden liegenden Patienten üblich, hinter dem Kopf des Patienten. Das zweite Crew-Mitglied befindet sich auf der rechten Patientenseite, appliziert die Narkose- und ggf. kreislaufstabilisierenden Medikamente, assistiert bei der Intubation mit Blick auf den Videobildschirm und hat die Absaugpumpe griffbereit zu seiner rechten Hand. Im Fall der Notwendigkeit eines alternativen Airway-Managements befindet sich das Material für die Koniotomie (Skalpell, Intubationskatheter) ebenfalls in der „Airway-Tasche“. Larynxmasken sind in einem Fach rechts neben dem Intubierenden aufbewahrt, das auch während des Flugs von beiden Teammitgliedern problemlos erreicht werden kann.

Für die sichere Durchführung sind gemäß der Erfahrung der Autoren folgende Aspekte elementar [17]:

- Auf diese Thematik fokussiertes Teamtraining unter realistischen Einsatzbedingungen (Lärm, Kommunikation über „Interkom“).
- Der i.v.-Zugang sollte nur bei guten Venenverhältnissen während des Flugs gelegt werden, da die Vibrationen während des Flugs die Punktion etwas erschweren. Bei unsicheren Venenverhältnissen sollte zumindest ein i.v.-Zugang vor Transportbeginn vorhanden sein.
- Es bedarf einer klaren „standard operating procedure“ (SOP) für die „in cabin RSI“.
- Evaluation des Atemwegs vor Transportbeginn: Bei Hinweisen auf eine möglicherweise erschwerte tracheale Intubation bzw. Videolaryngoskopie (z. B. schweres Mittelgesichtstrauma, schwere Blutung im Oropharynx, Obstruktion der oberen Atemwege) sollten die Narkoseeinleitung und das Airway-Management noch vor Beginn des Flugs erfolgen. Bei erschwerten Intubationsbedingungen kann so ggf. die Kabinentür für einen besseren Zugang zum Patienten geöffnet werden.
- Evaluation des Thorax vor Transportbeginn: Bei Hinweisen auf einen bereits bestehenden Pneumothorax sollte die weitere Stabilisierung zunächst vor Ort erfolgen.
- Positionierung der Teammitglieder: Das Airway-Management, also die Position am Kopfende, sollte von der in der trachealen Intubation erfahrensten Teammitglied eingenommen werden. Das zweite Teammitglied übernimmt die Position an der Seite des Patienten sowie die Applikation der Medikamente und die Kreislaufstabilisierung.
- Die Ablageorte des Materials zum Airway-Management und von Medikamenten müssen im Rahmen der SOP klar definiert sein. Aufgrund des reduzierten Platzangebots muss auf gute Ordnung geachtet werden.
- Zwingender Einsatz der Videolaryngoskopie und ausreichende Erfahrung darin.
- Da eine Auskultation während des Flugs unmöglich ist, sollten die Kontrolle der beidseitigen Ventilation und der Ausschluss eines Pneumothorax nach Beginn der mechanischen Ventilation sonographisch erfolgen. Der zwingende Einsatz der Kapnographie ist selbstverständlich.
- Falls sich ein Pneumothorax auf der nichtzugänglichen Patientenseite entwickelt und die Anlage einer Drainage in Bülau-Position unmöglich ist, erfolgt die Drainage in Monaldi-Position oder bei nur noch kurzer verbliebener Transportzeit die alleinige Nadeldekompression.

## Fazit für die Praxis

- In ausgewählten Fällen (intubationspflichtige Patienten mit zeitkritischen Verletzungen ohne zu erwartendes schwieriges Atemwegsmanagement) könnte zukünftig die Prähospitalzeit in der Luftrettung deutlich verkürzt werden, indem Narkoseeinleitung und Intubation in der Helikopterkabine und während des Transports ins Traumazentrum durchgeführt werden.
- Diese „in cabin rapid sequence induction“ sollte aber momentan nur durch zuvor trainierte Teams bei Vorliegen einer klaren „standard operating procedure“ (SOP) erfolgen.
